# Pathological Fracture in the Metatarsal Bone Due to an Intraosseous Schwannoma: A Case Report

**DOI:** 10.7759/cureus.95134

**Published:** 2025-10-22

**Authors:** Lu Shen, Lingling Song, Zhaoshu Huang, Yining Xiang, Deqing Song

**Affiliations:** 1 Radiology, The Affiliated Hospital of Guizhou Medical University, Guiyang, CHN; 2 Pathology, The Affiliated Hospital of Guizhou Medical University, Guiyang, CHN

**Keywords:** bone, computed tomography, foot, magnetic resonance imaging, schwannoma

## Abstract

Intraosseous schwannomas (IOSs) are uncommon benign tumors arising from nerve sheath cells. Schwannomas occurring in the metatarsal bones are extremely uncommon. This case report describes a young adult with a progressively enlarging mass in the foot, which led to limited mobility and ultimately resulted in a pathological fracture of the first metatarsal bone. Imaging studies revealed a diffuse, lytic lesion accompanied by surrounding soft tissue swelling. Histopathological evaluation confirmed the diagnosis of a schwannoma. Notably, this case highlights a key diagnostic challenge: its radiological and immunohistochemical features can be ambiguous. Although findings such as an elevated Ki-67 index suggested potential low-grade malignant transformation, retained H3K27me3 expression and an indolent clinical course confirmed the benign nature of the lesion. This case underscores the diagnostic complexity of ambiguous skeletal lesions and emphasizes the necessity of integrating clinical, radiological, and pathological assessments to achieve an accurate diagnosis and guide management. Reporting this case in the literature should help enhance awareness of rare variants of IOSs and advocate for the adoption of an integrated clinical-radiological-pathological workflow when managing ambiguous skeletal lesions.

## Introduction

Arising from nerve sheath cells, schwannomas (neurilemmomas) typically manifest as benign neoplasms. The predominant location for these neoplasms is head and neck soft tissues [1], as well as the spinal cord and spinal nerves [2], while their intraosseous variant represents an exceptionally uncommon entity [3]. Originating from Schwann cells within neural sheaths, this benign tumor exhibits distinctive histological characteristics. Skeletal manifestations of this pathology remain remarkably infrequent, constituting merely 0.1% to 0.2% of primary bone neoplasms [4,5]. It can affect virtually any bone, but the mandible and sacrum are the most frequently reported sites [3]. Additional documented anatomical sites encompass vertebral bodies, cranium, scapula, sternum, costal elements, metacarpal bones, femur, tibia, fibula, calcaneus, ulna, and phalangeal structures [4-7]. Schwannomas occurring in the metatarsal bones are extremely uncommon. 

The diagnosis of intraosseous schwannomas (IOSs) remains challenging due to their non-specific radiographic features, which often mimic more common benign bone lesions such as enchondroma, unicameral bone cysts, and giant cell tumors of bone [8]. This diagnostic ambiguity necessitates a high index of suspicion and underscores the importance of correlating imaging findings with histopathological and immunohistochemical analysis for a definitive diagnosis. In some cases, schwannomas may exhibit low-grade malignant features, which is extremely rare in clinical practice [9]. As a result, it may be challenging to differentiate schwannomas from malignant peripheral nerve sheath tumors [8]. The current study aims to describe the clinical and imaging findings, as well as histopathological aspects, of a case of a schwannoma in the first metatarsal bone that caused a pathological fracture. Another goal is to highlight these challenges, and this study aims to contribute to the recognition and management of this rare entity and provide a resource for clinical diagnosis and treatment.

## Case presentation

History

A 22-year-old woman presented with a one-year history of a self-palpated mass on the dorsal side of her right foot, which was approximately the size of a soybean, tender to pressure, and free of local skin redness, swelling, heat, or pain. She had not sought any medical intervention. Two months ago, the patient noticed that the mass on her right foot had significantly enlarged to approximately the size of an egg. It was firm in consistency, tender on palpation, without radiating pain, and without signs of local inflammation.

Laboratory investigation revealed the elevated alkaline phosphatase level of 134.20 U/L (normal range: 30.00-100.00 U/L) and the mildly decreased total osmolality of 275.4 mOsm/L (normal range: 280.00-320.00 mOsm/L).

Physical examination revealed a painful and swollen right first metatarsal bone with restricted mobility, as well as a firm mass of around 4×4 cm on the dorsum of the right foot. There were no signs of erythema, edema, increased local temperature, or spontaneous pain. Peripheral circulation to the right foot extremity was normal. The first toe had limited mobility, but the remaining four toes moved normally.

Imaging

**Figure 1:**
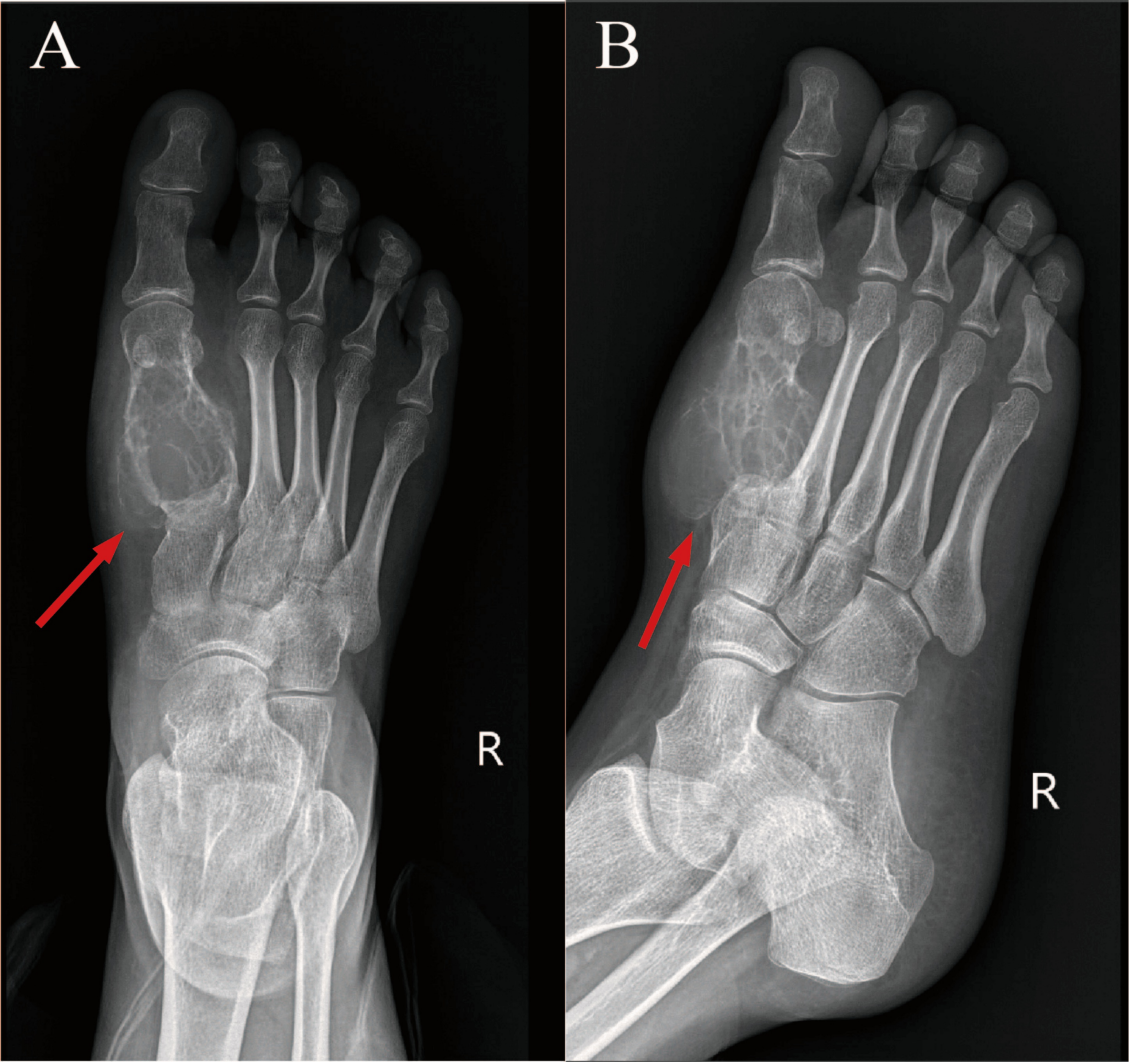
(A) AP and (B) lateral radiographs (A) AP and (B) lateral radiographs show an expansile and osteolytic bone destruction area in the right first metatarsal bone without an obvious periosteal reaction. The boundary with adjacent tissues remained clear, and there was swelling of the surrounding soft tissue.

Anteroposterior and oblique radiographs of the right foot demonstrated an expansile and osteolytic lesion in the right first metatarsal bone, approximately 35×36 mm in size, with several fine septations and trabecular remnants visible within the lesion. No significant periosteal reaction was observed. The lesion maintained well-defined borders with adjacent tissues, although surrounding soft tissue swelling was evident (Figure 1).

**Figure 2:**
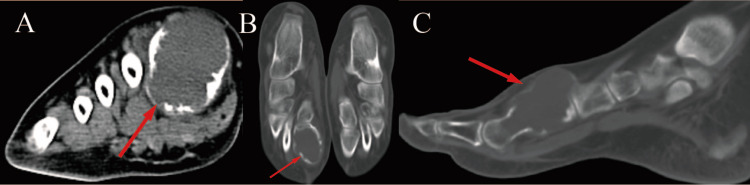
Preoperative CT scan of the feet with multiplanar reconstruction (MPR) Preoperative CT scan of the feet with multiplanar reconstruction (MPR) displays a pathological fracture traversing an expansile osteolytic process within the distal first metatarsal segment, exhibiting cortical expansion, endosteal scalloping, cortical attenuation, and an associated extraosseous soft tissue component.

Osseous and articular evaluation via three-dimensional computed tomography (3D-CT) demonstrated an expansile, osteolytic lesion within the right first metatarsal, with approximate dimensions of 35 mm×30 mm. The density was homogeneous, with a CT value of approximately 30 HU. Multiple fine septations and residual trabecular patterns were visible within the lesion. The cortical bone showed discontinuity, and the surrounding soft tissues were enlarged, with scattered areas of increased density. Initial orthopedic consultation occurred with a provisional diagnosis suggesting metatarsal enchondroma as the etiology for the pathological fracture. Further radiographic review revealed a large lesion of the first metatarsal shaft with some expansion of bone, endosteal scalloping, thinning of bone cortex, and accompanied by soft tissue mass (Figure 2).

**Figure 3:**
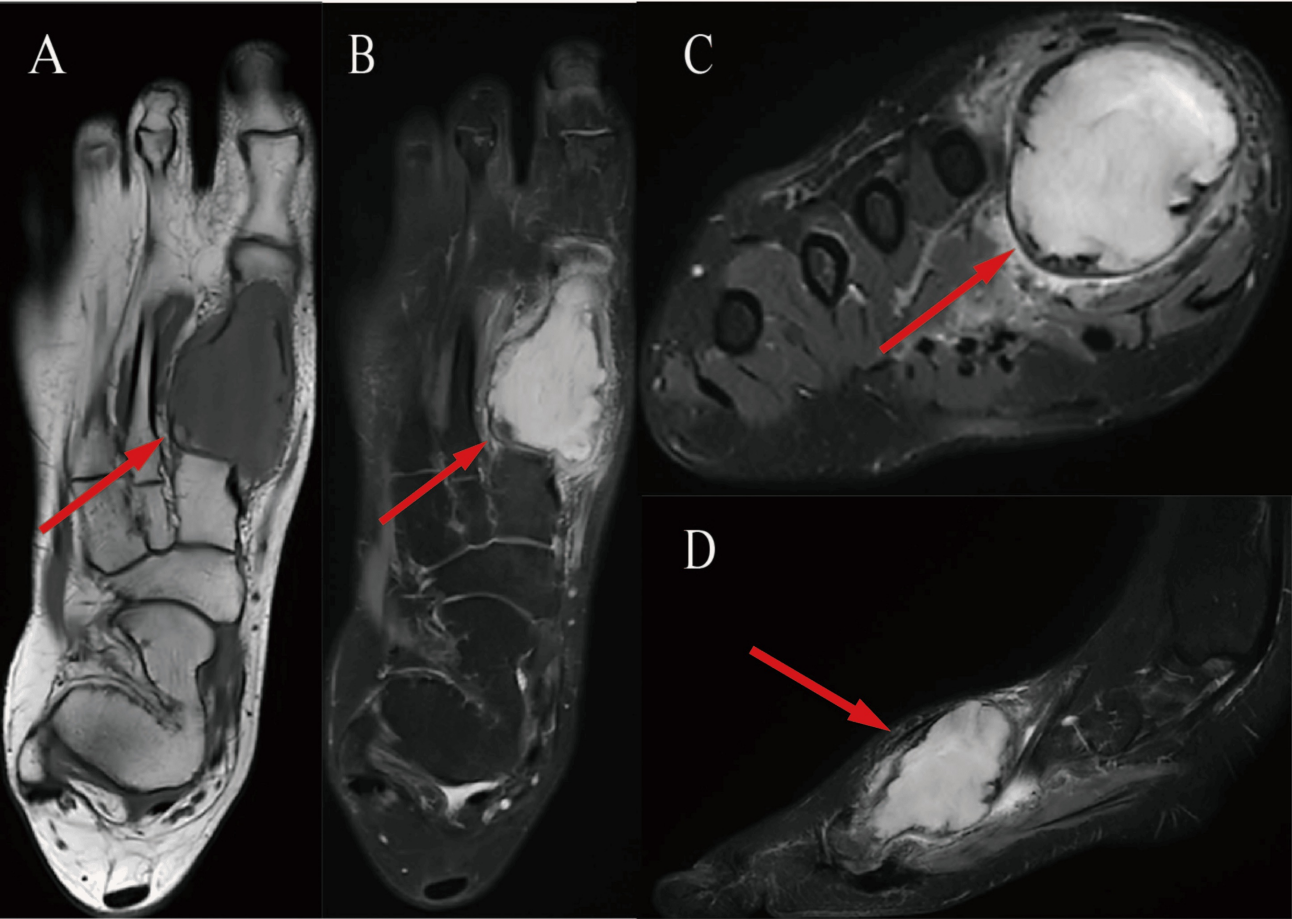
(A) Coronal T1-weigthted image, (B) coronal proton density weighted fat–saturated, (C) axial, and (D) sagittal T2-weighted fat–saturated MRI images (A) Coronal T1-weighted image, (B) coronal proton density weighted fat–saturated, (C) axial and (D) sagittal T2-weighted fat–saturated MRI images reveal a circumscribed neoplasm within the right first metatarsal bone, characterized by osseous expansion, cortical attenuation, and disruption of surrounding skeletal integrity. Signal characteristics include relative hypointensity on T1-weighted sequences and a pronounced hyperintense signal pattern on T2-weighted acquisitions.

Magnetic resonance imaging (MRI) of right foot demonstrated a mass measuring 49mm×29mm×34mm exhibiting mild hypointensity on T1-weighted sequences and marked hyperintensity on T2-weighted acquisitions. This lesion displayed well-defined margins against surrounding structures, alongside adjacent cortical attenuation and interruption of osseous continuity (Figure 3).

Pathology

**Figure 4:**
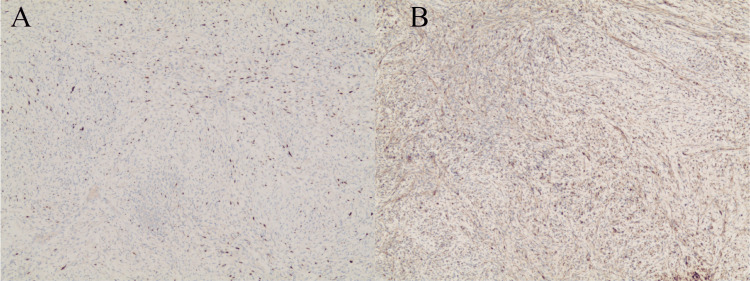
(A) Low-power view with SOX-10 and (B) S-100 stains (A) Low-power view with SOX-10 and B S-100 stains highlighting the characteristic schwannoma architecture featuring whorled formations and intersecting fascicles of neoplastic spindle cells with distinctive nuclear palisading [10 ×].

Pathological examination revealed typical schwannoma morphology (Figure 4).

Immunohistochemical profiling demonstrated strong reactivity for S-100 protein, SOX-10, CD68, and H3K27me3 (focally attenuated in certain regions), whereas neoplastic cells exhibited complete absence of immunoreactivity for CK, EMA, desmin, SMA, Caldesmon, Myogenin, DOG-1, STAT6, and MUC-4. Proliferative activity assessment using the Ki-67 labeling index revealed approximately 20% positive nuclear staining.

During the follow-up period, the patient experienced no complications. At the six-month follow-up assessment, the metatarsal region exhibited a full and pain-free range of motion. Informed consent was obtained from the patient.

## Discussion

Schwannomas represent benign peripheral nerve sheath neoplasms derived from Schwann cell proliferation. They are characterized by slow growth, localized symptoms such as pain and swelling, and potential neurological deterioration due to structural damage, axonolysis, and Wallerian degeneration [10]. Two important characteristics may make diagnosing metatarsal schwannomas difficult: their radiological resemblance to common cystic or fibrocystic bone lesions and their remarkably low rates of malignancy [8]. We synthesized our findings with previously reported cases in the literature to better characterize the radiologic and clinical presentation of IOSs, as summarized in Table 1. Clinically, IOSs are predominantly benign neoplasms, typically identified incidentally during radiographic evaluation or diagnosed following investigation of symptoms such as pain or swelling. When symptomatic, the onset is characteristically insidious, with symptoms frequently preceding the definitive diagnosis by several years. This clinical presentation concurs with that observed in our case and aligns with the descriptions documented in the cases summarized within Table 1.

**Table 1:** Review of published cases of intraosseous schwannoma in extremities N/A: not available, T1WI: T1-weighted imaging, T2WI: T2-weighted imaging.

Author, year	Age, years	Gender	Site	Clinical Presentation	Imaging Findings	Immunohistochemistry
Huajun et al., 2021, [4]	55	Female	Right proximal humerus	Pain, swelling, ecchymosis, and pathologic fracture	Radiographs showed a well-defined, osteolytic lesion with endosteal scalloping and trabeculation in the proximal humerus, without periosteal reaction, soft tissue mass, or calcifications. On MRI, the lesion showed cortical invasion and associated edema, appearing isointense on T1WI and hyperintense on T2WI.	Typical S100 + and SOX-10+
Meek et al., 2007, [8]	27	Female	Left first metatarsal	Pain, swelling, and pathologic fracture	Radiographs showed a pathologic fracture through an expanding lytic lesion involving the distal half of the first metatarsal. MRI demonstrated an amorphous mass occupying the medullary canal of the bone, but with a breach of the plantar aspect of the cortex with apparent localized bony destruction.	Typical S100 +
Wang et al., 2016, [11]	50	Female	Right tarsal navicular, cuboid, all 3 cuneiforms, and the second and third metatarsal bases	Pain and swelling	Radiographs revealed a large, well-defined osteolytic lesion with endosteal scalloping and trabeculated contours at the margins. MRI revealed an isointense signal to skeletal muscle on T1WI and a hyperintense signal to subcutaneous fat on T2WI.	Typical S100 +
Ansari et al., 2014, [12]	48	Female	Right first metatarsal	Pain and swelling	Radiographic views of the foot showing an expansile, osteolytic benign lesion involving the first metatarsal. MRI showed an expansile and heterogeneous isointense lesion on T1WI and the lesion appeared to be heterogeneous and hyperintense on T2WI.	N/A

A study by Carvajal et al. found that women are more susceptible to developing peripheral nerve sheath tumors (PNSTs) [13]. Andrea Angelini's research demonstrated that schwannomas affect men and women equally, and the age group with the highest incidence rate is 30 - 40 years old [14]. The patient in our case is a 22-year-old female. Trauma, Carney syndrome, and type 1 or type 2 neurofibromatosis (NF) may play a causal role in the etiology of these tumors [14,15].

MRI plays an important role in determining the extent of deep schwannomas and identifying the nerve of origin [3,16]. IOSs exhibit benign, low-grade malignant, or indolent imaging features. Conventional radiography and computed tomography typically demonstrate predominantly osteolytic lesions characterized by narrow transitional zones, thin peripheral sclerotic margins, varying degrees of osseous expansion, and minimal periosteal response. Cortical disruption and soft tissue extension occur in 40% of IOSs, but this is uncommon [3]. Relative to skeletal musculature, schwannomas typically demonstrate signal characteristics ranging from isointensity to mild hypointensity on T1-weighted sequences, juxtaposed with heterogeneous hyperintense signal patterns on T2-weighted acquisitions [3,14]. Our case demonstrated MRI signal intensities similar to those of the cases summarized in Table 1. Following gadolinium administration, these neoplasms exhibit variable contrast enhancement patterns [3,6]. Characteristic radiographic findings may include the "fascicular sign," "target sign," and "fat split sign," although it should be noted that these features are not pathognomonic and may occasionally be observed in neurofibromas. The pathological basis for the distinctive "target" sign appreciated on T2-weighted imaging in soft tissue schwannomas corresponds to the architectural arrangement of centrally located hypercellular Antoni A regions surrounded by peripherally distributed hypocellular Antoni B components [3]. Detailed evaluation of lesional margins and peritumoral characteristics facilitates the distinction between large heterogeneous schwannomas and malignant PNSTs on magnetic resonance studies. The presence of fat split sign and bright rim sign, coupled with the absence of lobulated morphology and extensive edema, aids in the diagnosis of schwannomas [17]. However, the characteristic MRI signal intensity pattern of soft tissue schwannoma was not observed in our case and few reported cases in the literature have described such a pattern [1].

The differential diagnosis of metatarsal nerve schwannomas encompasses several osseous entities, such as enchondroma, unicameral bone cyst, aneurysmal bone cyst, chondroblastoma, non-ossifying fibroma, and giant cell tumor, presenting significant diagnostic challenges [5,8,16]. Accurate diagnosis necessitates an integrated assessment of radiographic features correlated with the comprehensive clinical context to facilitate appropriate diagnostic discrimination. Enchondroma is a well-defined lytic lesion that typically occurs in the short tubular bones of the hand. It may be accompanied by punctate or annular calcification, cortical thinning and expansion, and other characteristic features. A solitary bone cyst typically presents as a unilocular cystic lesion in the metaphysis of adolescents, usually without significant periosteal reaction. An aneurysmal bone cyst presents as an eccentric, expansile, well-defined lytic lesion, often accompanied by periosteal reaction and characteristic fluid-fluid levels on imaging. Benign chondroblastoma typically occurs in the epiphyses of young patients and is characterized by the central osteolytic destruction surrounded by a thin sclerotic rim. Non-ossifying fibroma is generally asymptomatic and more prevalent in young people. Radiographs typically demonstrate eccentric metaphyseal lytic lesions with surrounding sclerosis and no obvious periosteal reaction. Giant cell tumor typically occurs in middle-aged women and radiographically presents as an eccentric, lytic lesion in the epiphysis, often with a characteristic "soap-bubble" appearance [3,8].

Histopathologically, schwannomas demonstrate characteristic biphasic architecture with Antoni A and B regions. Antoni A areas contain densely packed spindle cells arranged in fascicles/lobules, displaying serpentine nuclei and indistinct cytoplasmic borders [16]. Mitotic figures are rare. Pathognomonic Verocay bodies exhibit nuclear palisading flanking anuclear eosinophilic zones.

The immunohistochemical profile provides critical diagnostic clues. All variants of schwannoma demonstrate diffuse and strong positivity for S100 and SOX10. However, a malignant peripheral nerve sheath tumor (MPNST) is mostly negative for S100 protein and SOX10 and is seldom strongly and diffusely positive. In our case, focal positivity for S-100 and SOX-10 was observed. In schwannomas, Ki-67 is typically <5%, yet in our case, it markedly exceeded this range (>20%). Additionally, CD34 demonstrates diffuse expression in schwannoma but complete loss in the MPNST [18]. However, our case demonstrated partial loss of CD34 expression. Crucially, the focal (not complete) loss of H3K27me3 expression argues against MPNST, where these markers often show diffuse abnormalities [18,19]. We summarize the histological changes and expression changes of typical marker proteins in schwannomas and MPNST in Table 2. Therefore, despite these atypical features, the overall histopathological and immunophenotypic profile supports a diagnosis of benign schwannoma with unusual immunohistochemical features, rather than overt malignancy or a borderline lesion.

**Table 2:** Histological features of the schwannoma, MPNST, and our case +++: diffusely and strongly positive expression; +: focally attenuated in certain regions; -: very low or no expression MPNST: Malignant peripheral nerve sheath tumor

	Expression of Marker Proteins			
	S100/SOX10	Ki-67	CD34	H3K27me3
Schwannoma	+~~+++	<5%	+~+++	+~+++
MPNST	-	>10%	-	-
Our Case	+	>20%	+	+

The management of PNSTs primarily hinges on complete surgical resection with wide negative margins, which represents the cornerstone of treatment and the most significant prognostic factor for local control and overall survival. The prognosis is largely determined by the tumor location, underlying genetic predispositions such as NF3, and the status of the surgical margins. Consequently, long-term postoperative surveillance is essential for detecting potential recurrence [20].

## Conclusions

This case underscores a key diagnostic challenge: its radiological and immunohistochemical features can be ambiguous. While findings like an elevated Ki-67 index raised diagnostic consideration, the retained H3K27me3 expression and indolent clinical course confirmed a benign diagnosis. The definitive diagnosis for such rare lesions relies on a multidisciplinary synthesis of clinical, radiological, and pathological data. This integrated approach is essential to avoid misdiagnosis and guide appropriate management.
